# Polymer‐Based Multiparameter Sensing Integrated Photonic Chip for Health Monitoring

**DOI:** 10.1002/advs.76123

**Published:** 2026-06-12

**Authors:** Xiaolin Li, Hongqiang Li, Ming Han, Xin Liu, Mingjun Ding, Ziying Niu, Dongyang Liu, Liyuan Yang, Lizhen Zhang, Francisco Hernández‐Ramírez, Lu Cao, Enbang Li

**Affiliations:** ^1^ Tianjin Key Laboratory of Optoelectronic Detection Technology and Systems School of Electronics and Information Engineering Tiangong University Tianjin China; ^2^ Shaoxing Keqiao Research Institute of Tiangong University Shaoxing China; ^3^ Tianjin Key Laboratory of Intelligent Control of Electrical Equipment School of Control Science and Engineering Tiangong University Tianjin China; ^4^ Engineering Teaching Practice Training Center Tiangong University Tianjin China; ^5^ School of Chemical Engineering and Technology Tiangong University Tianjin China; ^6^ School of Innovation and Entrepreneurship Tiangong University Tianjin China; ^7^ Department of Electronics and Biomedical Engineering Universitat de Barcelona Barcelona Spain; ^8^ Worldsensing Barcelona Spain; ^9^ Chest Hospital Tianjin University Tianjin China; ^10^ Center for Medical Radiation Physics University of Wollongong Wollongong New South Wales Australia; ^11^ School of Physics Faculty of Engineering and Information Sciences University of Wollongong Wollongong New South Wales Australia

**Keywords:** blood glucose, electrocardiogram, integrated photonic chip, Mach–Zehnder modulator, pressure, temperature, waveguide Bragg grating

## Abstract

Optical sensors are essential in medical diagnostics and detect variations in light intensity, phase, wavelength, polarization, or interference. However, conventional devices face limitations in multiplexing capability, surface functionalization, and integration. Here, we propose a monolithic integrated poly(dimethylsiloxane) (PDMS)/poly(methyl methacrylate) (PMMA) photonic chip that monolithically integrates two photonic waveguide Bragg grating (WBG) blood glucose sensors, a photonic WBG pressure sensor array, four photonic WBG temperature sensors, a photonic Mach–Zehnder modulator (MZM) electrocardiogram (ECG) sensor, and a photonic Mach–Zehnder interferometer (MZI) sweat glucose sensor, together with an on–chip arrayed waveguide grating (AWG) demodulation unit. The experimental results demonstrate that the WBG glucose sensor operates stably from 0 to 2.3 mg/mL, the pressure sensor from 0 to 12 kPa, and the temperature sensor from 35 to 41°C. The MZM ECG sensor achieves a half–wave voltage of 1.6 V, with performance comparable to that of commercial systems. The MZI sweat glucose sensor offers a detection range of 0–0.2 mg/mL, a sensitivity of approximately 9.3595 mW/(mg/mL), and high linearity. With a miniature footprint of approximately 1 cm × 0.5 cm, this work offers a robust on‐chip multiparameter sensing solution, paving the way for next–generation wearable continuous health monitoring.

## Graphical abstract

A monolithic integrated photonic chip simultaneously measures blood glucose, pressure, temperature, ECG, and sweat glucose. Combining multiple Bragg grating sensors, Mach–Zehnder modulators, and an on–‐chip demodulation unit, this compact chip (1 cm × 0.5 cm) offers robust multiparameter detection. These results pave the way for next‐generation wearable continuous health monitoring.

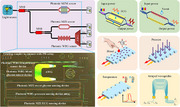

## 1 Introduction

Health monitoring involves the continuous tracking of various health‐related aspects. This process involves measuring parameters such as body temperature, pulse, electrocardiogram (ECG) parameters, and blood glucose [1, 2, 3, 4]. Health monitoring involves real‐time online monitoring of the health conditions of users and helps doctors detect diseases early, which is crucial for maintaining good health. Existing measurement methods rely primarily on independent machines such as thermometers [5, 6], ECG monitors [7, 8], blood glucose meters [9, 10], and blood pressure monitors [11, 12, 13]. Although these machines provide relatively accurate measurements, they are often bulky and single‐function, making it challenging to simultaneously monitor multiple parameters encompassing both vital signs and metabolic indicators [14, 15]. This limitation not only restricts the overall assessment of the health status of an individual but also reduces the efficiency of early detection and warning of potential diseases [16, 17, 18, 19, 20].

Optical sensors, powered by photonics, are integral to modern technology and provide precise measurements, real‐time monitoring, and advanced new possibilities for integrated multiparameter health monitoring [21, 22, 23]. Compared with sensors that are based on electrical principles, optical sensors offer unique advantages, including significant immunity to electromagnetic interference, high detection accuracy, high response speed, and the ability to acquire multidimensional information [24, 25, 26, 27, 28, 29, 30]. Additionally, the integration of optical waveguides with microfluidic technologies has created new opportunities for measuring biochemical indicators in body fluids [31, 32, 33, 34], facilitating the simultaneous monitoring of physiological and biochemical signals on a single platform [35, 36]. However, most existing optical wearable sensors remain limited to single‐function sensors and fail to overcome key challenges such as insufficient sensitivity, limited device flexibility, poor skin conformability, and difficulties in large–scale integration [37, 38, 39].

By integrating multiple optical functionalities onto a single chip, an integrated photonic chip fabricated from polymer materials (poly (dimethylsiloxane) (PDMS)/poly (methylmethacrylate) (PMMA)) is proposed in this study. The photonic chip includes two photonic waveguide Bragg grating (WBG) blood glucose sensors, a photonic WBG pressure sensor array, four photonic WBG temperature sensors, a photonic Mach–Zehnder modulator (MZM) ECG sensor, a photonic Mach–Zehnder interferometer (MZI) sweat glucose sensor, and an on–chip arrayed waveguide grating (AWG) demodulation unit. By matching multiple wavelength channels and optimizing the optical path, the AWG is used to demodulate the wavelength of the WBG sensors, and the chip facilitates multiparameter sensing of blood glucose, pulse, body temperature, ECG signals, and sweat glucose. This work aimed to provide system‐level integration solutions and key technical references for integrated photonic chips for wearable health monitoring.

## 2 Results

### 2.1 Integrated Photonic Chip Concept

**FIGURE 1:**
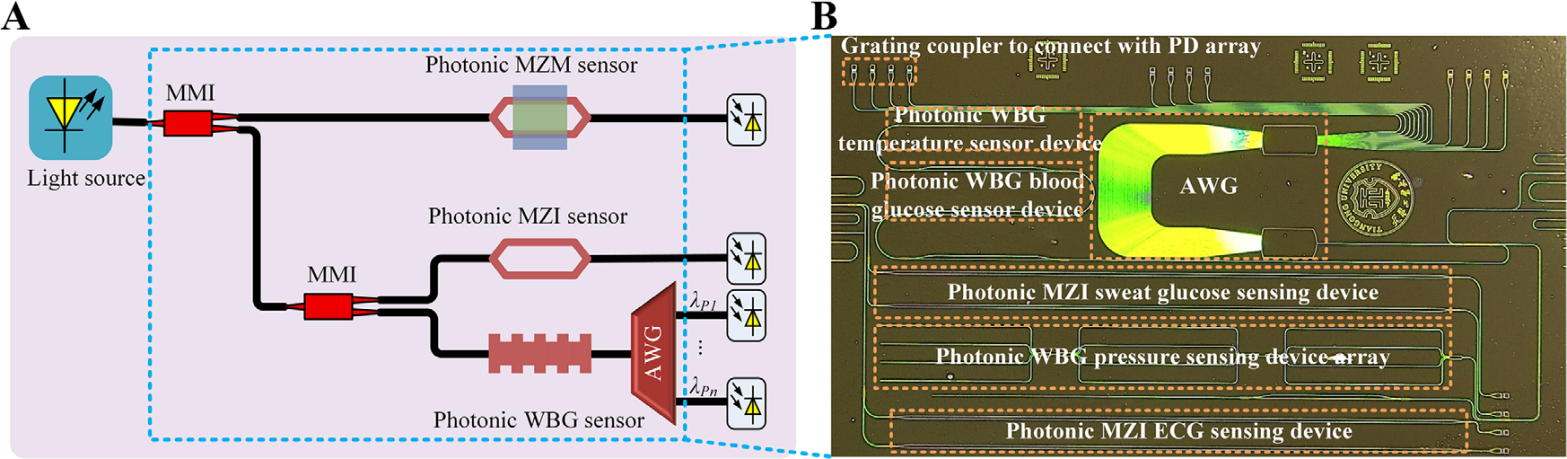
Schematic illustration and layout of the polymer‐integrated photonic chip. (A) Overview of the integrated photonic chip. (B) Optical microscopy image of the fabricated chip.

An integrated photonic chip incorporates multiple sensing units to enable simultaneous multiparameter measurement. The chip designed herein comprises photonic WBG glucose sensors, a photonic WBG pressure sensor array, a photonic WBG temperature sensor, a photonic MZM ECG sensor, a photonic MZI sweat glucose sensor, and together with an AWG unit, curved waveguides, multimode interference (MMI) couplers, and input/output grating couplers for optical coupling (Figure 1A). The photonic MZI sweat glucose sensor and photonic MZM ECG sensor generate intensity‐modulated outputs that can be directly measured through the grating couplers, whereas the photonic WBG sensors encode external stimuli through shifts in their reflected center wavelengths. The waveguide structure comprises 10 µm thick upper and lower PDMS claddings and a 1‐µm PMMA core, with a 200‐nm gold layer deposited on the upper PDMS cladding to provide electrical tuning. To enhance the interaction between the guided optical field and the analytes, the upper claddings in the sensing regions of the photonic WBG glucose sensors and photonic MZI sweat glucose sensor are locally etched. A microscopy image of the proposed photonic integrated chip is shown in Figure 1B.

### 2.2 Photonic WBG Blood Glucose Sensor Design and Characterization

**FIGURE 2:**
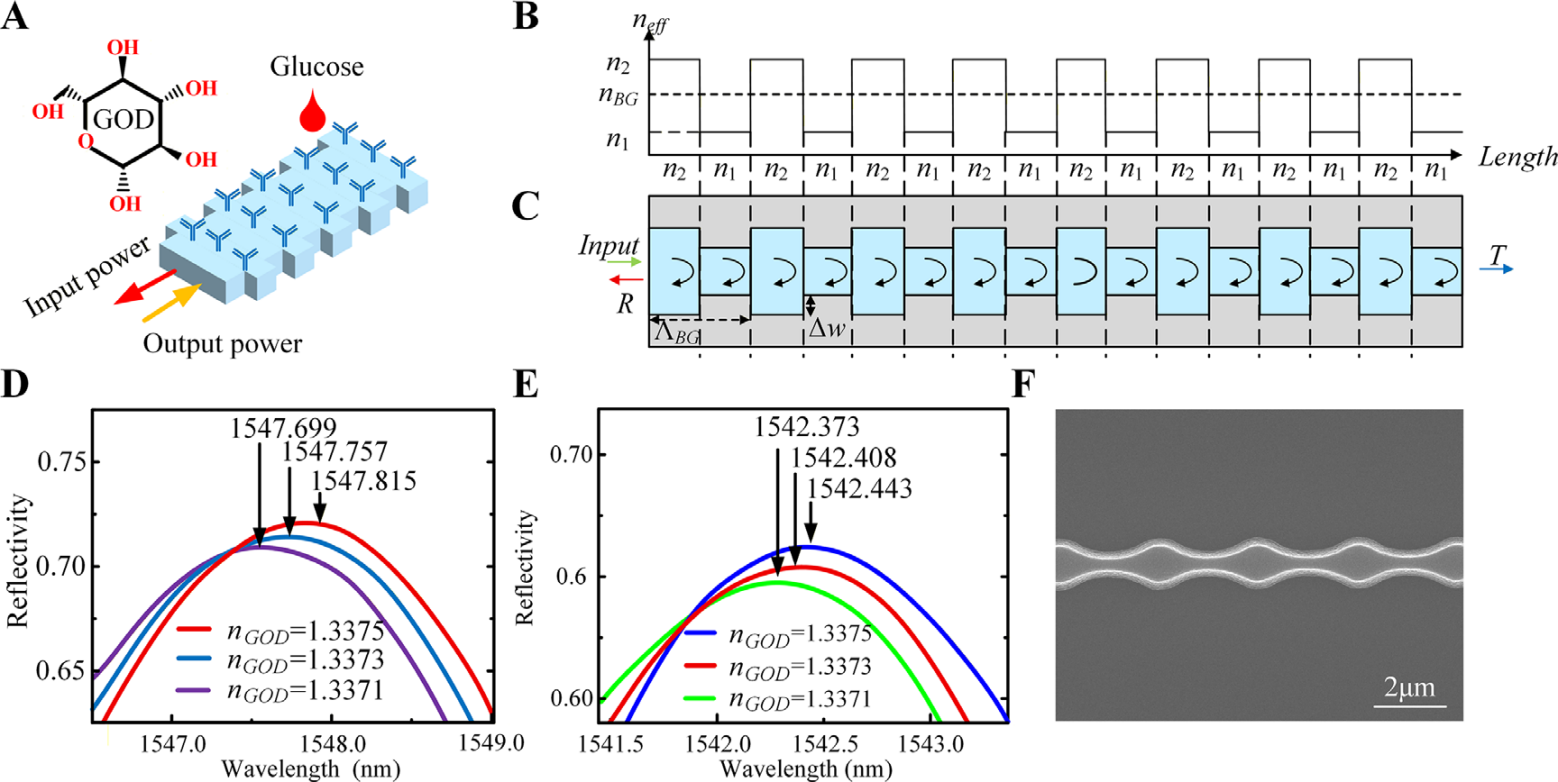
Simulation results and SEM image of the photonic WBG blood glucose sensor. (A) Schematic illustration of the photonic WBG blood glucose sensors. (B) Longitudinal effective index profile of a sidewall grating; *n_2_*: high effective index, *n_1_*: low effective index, *n_BG_*: effective index of the WBG. (C) Uniform WBG. (D) Simulation results showing the relationship between the central wavelength of WBG_1_ and changes in the glucose concentration. (E) Simulation results showing the relationship between the central wavelength of WBG_2_ and changes in the glucose concentration. (F) Overall view of the fabricated WBG blood glucose sensor.

The implementation of the blood glucose WBG sensor involves the periodic modulation of the waveguide geometry to adjust the effective index, as shown in Figure 2A. Input light is reflected at each boundary, and the phase is determined by the wavelength and period of the photonic WBG blood glucose sensor (denoted as *Λ_WBG_*). When the phase matching condition, i.e., *λ_WBG_* = 2*n_BG_Λ_BG_*, is satisfied, the reflected optical mode is constructed around *λ_WBG_*, where *λ_WBG_* is the central wavelength of the reflected light (also referred to as the Bragg wavelength), and *n_BG_* denotes the effective index of the photonic WBG blood glucose sensor (Figure 2B). When the blood glucose solution flows through this window, part of the evanescent field penetrates the sensitive layer and interacts with the glucose solution, thereby causing a change in the effective refractive index of the waveguide. This change results in a shift in the central wavelength of the reflected signal.

To achieve blood glucose selectivity, glucose oxidase (GOD) is immobilized in the window area as a specific recognition enzyme. When blood glucose molecules diffuse into the sensitive layer, an enzymatic oxidation reaction occurs, thereby causing changes in the concentration and refractive index of the local solution. These changes, in turn, enhance the response of the WBG reflection spectrum to the blood glucose concentration. To improve detection reliability and adapt to a single‐channel demodulation architecture, we designed two parameter‐differentiated redundant photonic WBG blood glucose sensors (Figure 2C). The design parameters for blood glucose WBG_1_ are as follows: a central wavelength of 1552 nm, a grating period of 2700 nm, a waveguide width of 2500 nm, a modulation depth of 500 nm, and a maximum reflectivity of 0.75. As shown in Figure 2D, when the refractive index changed from 1.3371 to 1.3375, the central wavelength of the photonic WBG_1_ blood glucose sensor decreased from 1547.815 to 1547.699 nm, corresponding to a blood glucose sensitivity of 290 nm/refractive index unit (RIU). The determined parameters for blood glucose WBG_2_ are as follows: a central wavelength of 1546 nm, a grating period of 2726 nm, a waveguide width of 2500 nm, a modulation depth of 690 nm, and a maximum reflectivity of 0.7. As shown in Figure 2E, under the same refractive index change, the central wavelength of the photonic WBG_2_ blood glucose sensor decreased from 1542.443 to 1542.373 nm, corresponding to a blood glucose sensitivity of 175 nm/RIU. The two sensors are demodulated through different AWG channels, which can effectively avoid detection failure caused by fabrication fluctuations. The simulation results and a scanning electron microscopy (SEM) image are shown in Figure 2F.

### 2.3 Photonic WBG Pressure Sensor Design and Characterization

**FIGURE 3:**
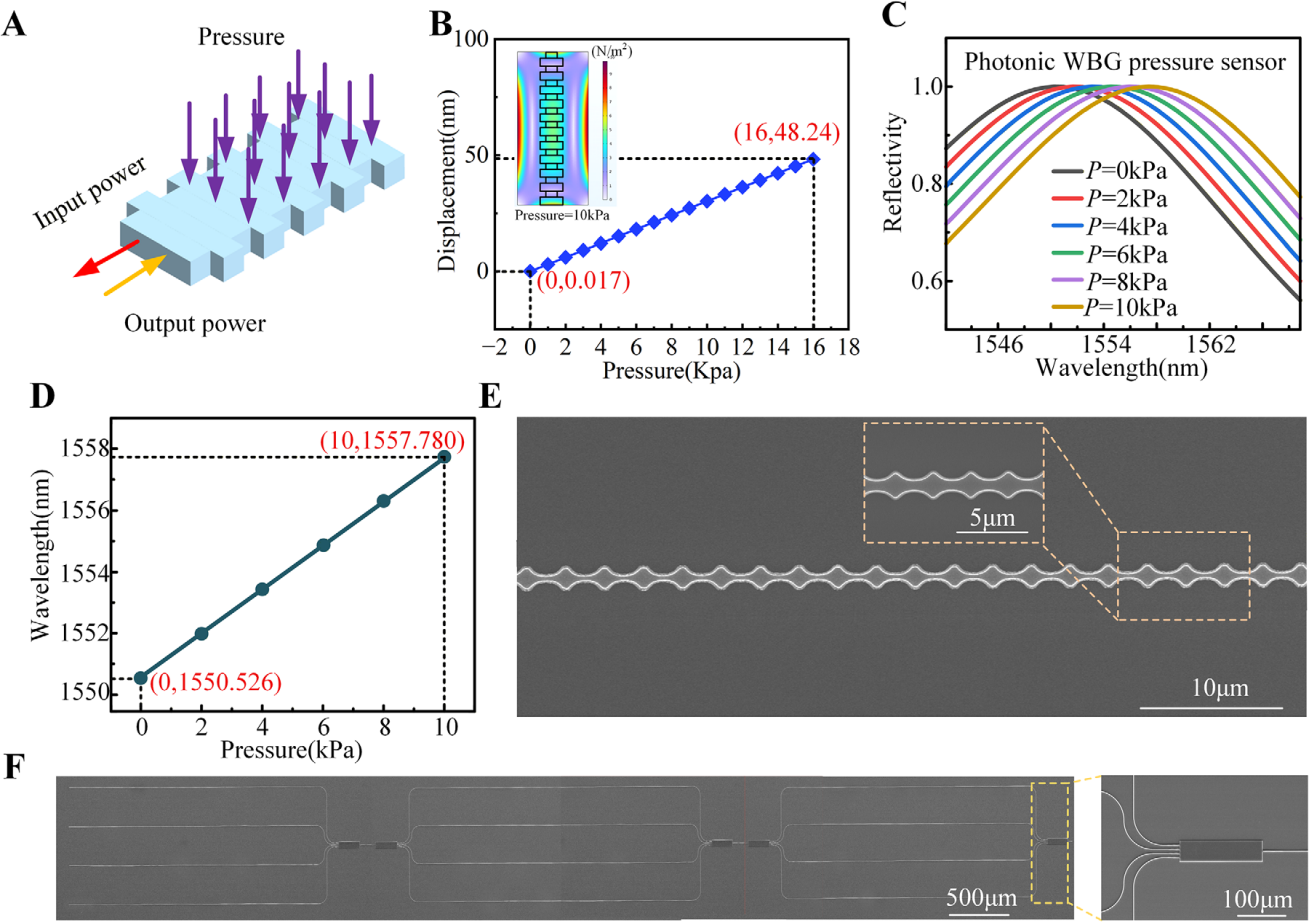
Simulation results and SEM images of the photonic WBG pressure sensor. (A) Schematic illustration of the photonic WBG pressure sensors. (B) Relationship between deformation and pressure. (C) Shift in the central wavelength of the WBG under different pressures. (D) Relationship between the central wavelength of the WBG and pressure changes. (E) Overall view of the fabricated WBG pressure sensor. The inset shows the magnified section. (F) Overall SEM image of the photonic WBG pressure sensor.

Photonic WBG pressure sensors measure pressure by leveraging the sensitivity of Bragg reflection to waveguide deformation and refractive index changes. When external pressure is applied to the WBG surface, the waveguide structure experiences slight mechanical deformation (Figure 3A). On the one hand, the change in the physical dimensions of the waveguide causes a change in the grating period (*Λ*). On the other hand, the photoelastic effect induced by stress alters the effective refractive index (*n_eff_*) of the waveguide. The combined effect of these changes results in a shift in the central wavelength of the Bragg reflection, enabling quantitative measurement of the external pressure by monitoring the wavelength shift.

The variation in the refractive index induced by pressure can be quantitatively described by the photoelastic effect following Δ*n = KP*, where the photoelastic coefficient is expressed as *K = n^3^(1−2µ)/(2E)*. With the PMMA core parameters of *n≈*1.488, *µ≈*0.35, and *E≈*2.5 GPa, the calculated *K* was approximately 1.27 × 10^−8^Pa^−1^. In the range of 0–10 kPa, the pressure–induced refractive index change was Δ*n≈*1.27 × 10^−10^, which is much smaller than the effective refractive index variation caused by periodic deformation (Δ*neff≈*0.0015). Thus, the wavelength shift is dominated mainly by mechanical deformation rather than photoelastic modulation. A coupled mechanical–optical simulation was implemented in this study. A 21 µm‐thick PDMS/PMMA composite film model was constructed in COMSOL. Based on the three‐dimensional elastic theory *σ = T·ε*, the structural deformation along the grating periodic direction was solved, and a linear relationship between the effective deformation and pressure was obtained: *D* = *3.01466P* + *0.00375*, indicating a period deformation of 3.01466 nm under a pressure of 1 kPa. (Figure 3B).

A single photonic WBG pressure sensor was first designed, and the parameters were as follows: a period of 2642 nm, a waveguide width of 3000 nm, a modulation depth of 600 nm, a wavelength range of 1518–1580 nm, and a maximum reflectivity of 0.9. When the pressure changed from 0 to 10 kPa, the central wavelength of the photonic WBG pressure sensor increased from 1550.526 to 1557.780 nm. The fitting curve for the pressure was *y =* 0.7077x + 1550.7598, with a sensitivity of 0.7077 nm/kPa and *R^2^* = 0.99621. (Figure 3C,D). SEM images of the photonic WBG pressure sensor are shown in Figure 3E. To address the limitations of a single pressure sensor, namely, its ability to withstand only a small range of pressure changes in practical applications and its localized response, which restricts the sensing area, the single photonic WBG pressure sensor was expanded into a 4 × 3 WBG array structure in this study (Figure 3E). The proposed sensor array has a compact footprint of 8 mm × 750 µm (Figure 3F). The arrayed design increases the effective pressure‐sensing area, thereby increasing the overall sensitivity and signal intensity.

### 2.4 Photonic WBG Temperature Sensor Design and Characterization

**FIGURE 4:**
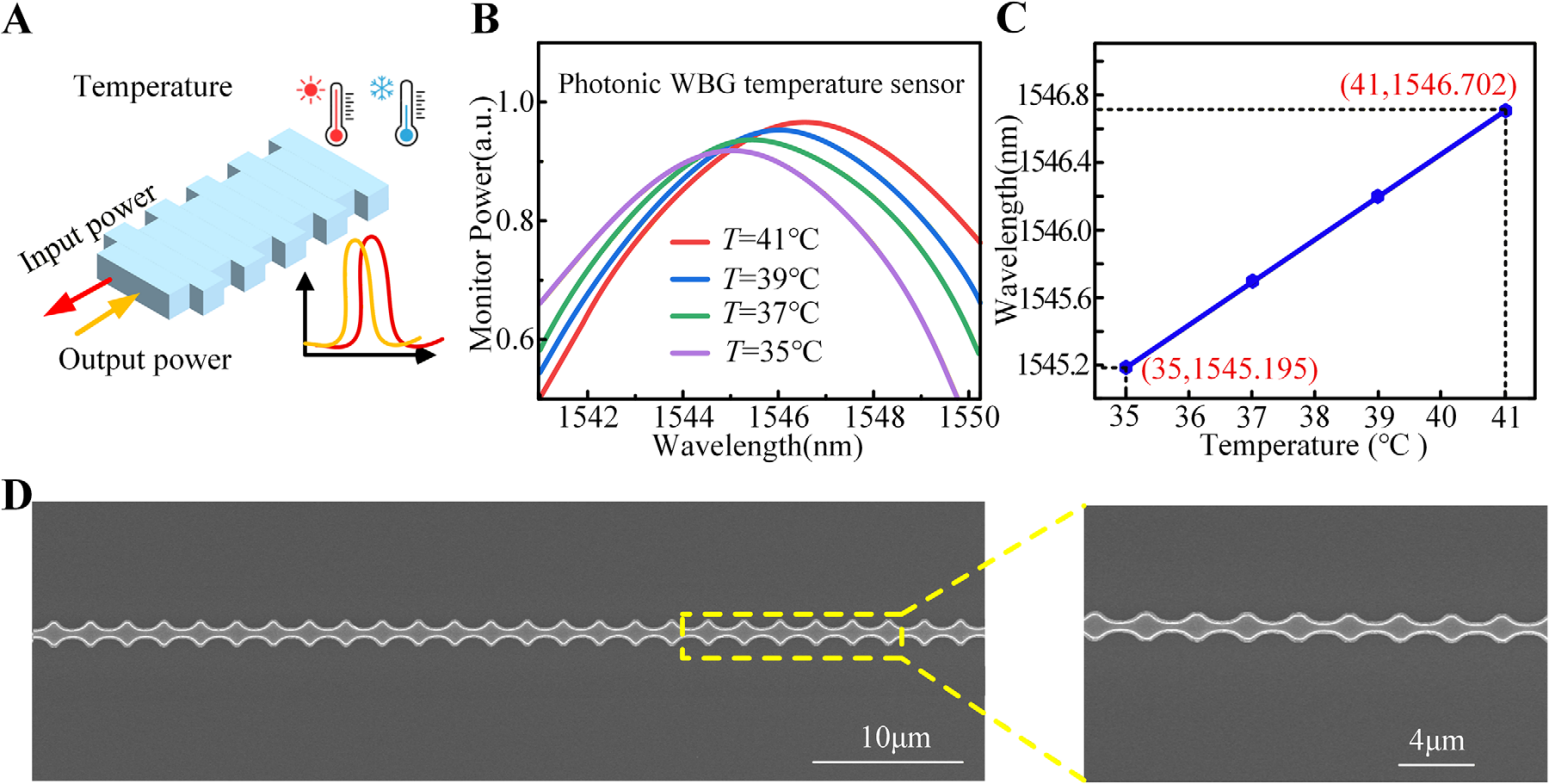
Simulation results and SEM images of the photonic WBG temperature sensor. (A) Schematic illustration of the photonic WBG temperature sensors. (B) Shift in the central wavelength of WBG at different temperatures. (C) Relationship between the central wavelength of WBG and temperature changes. (D) Overall view of the fabricated WBG temperature sensor. The inset shows the magnified section.

As the temperature changes, the photonic WBG temperature sensor operates on the basis of the Bragg reflection effect, and two effects occur. First, the refractive index of the waveguide material varies because of the thermo–optic effect. Second, the grating period changes because of thermal expansion. The combined influence of these effects causes a shift in the Bragg central wavelength, with the magnitude of this shift approximately linearly related to the change in temperature, thus enabling temperature measurement (Figure 4A).

The four proposed photonic WBG temperature sensors share identical structural parameters except for the grating period number, which is tailored to adjust their central operating wavelengths. The grating period numbers were set to 1000, 850, 833, and 805, corresponding to central wavelengths of 1550, 1547, 1544, and 1542 nm, respectively. The final parameters for the photonic WBG temperature sensor were as follows: a grating period of 2000 nm, a waveguide width of 1700 nm, a modulation depth of 200 nm, and a maximum reflectivity of 0.9. When the temperature changed from 35°C to 41°C, the central wavelength of the photonic WBG temperature sensor increased from 1545.195 to 1546.702 nm. The temperature fitting equation was *y =* 0.25419x + 1536.28385, with a temperature sensitivity of 254.19 pm/°C and a coefficient of determination R^2^ = 0.99998 (Figure 4B,C). The SEM image of the photonic WBG temperature sensor is shown in Figure 4D.

### 2.5 Photonic MZM ECG Sensor Design and Characterization

**FIGURE 5:**
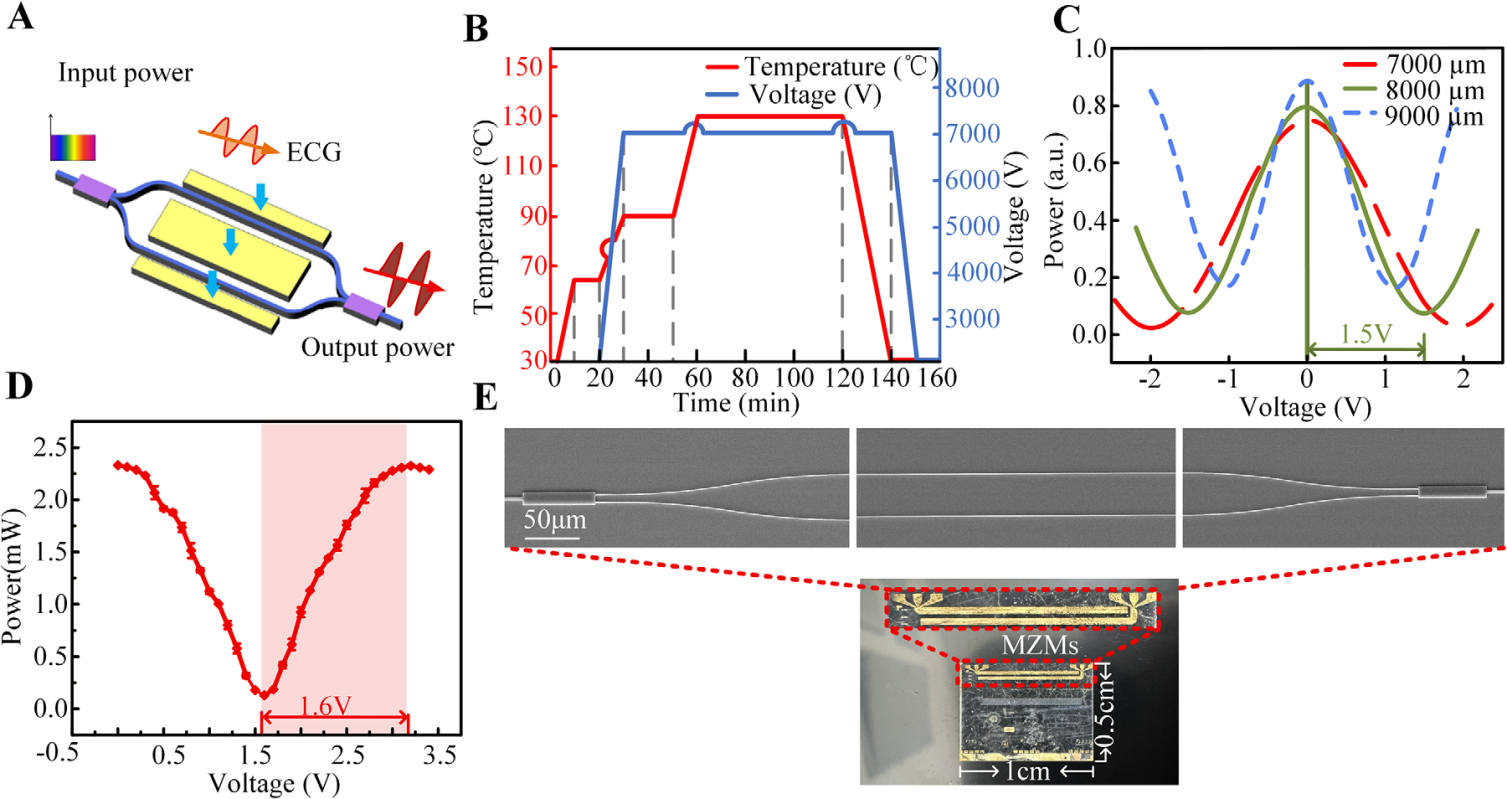
Schematic of the photonic MZM ECG sensor and SEM images of the fabricated device. (A) Schematic illustration of the photonic MZM ECG sensor. (B) Control curve of the polymer film polarization voltage and polarization temperature. (C) Relationship between the arm length and output power. (D) Relationship between the voltage and output power. (E) SEM image and sample chip image of the photonic MZM ECG sensor.

As shown in Figure 5A, the photonic MZM ECG sensor comprises an input MMI coupler, bent waveguides, modulation arms, an output MMI coupler, and parallel electrodes. In this study, the electro–optic polymer is prepared by mixing the CLD‐1 chromophore and PMMA at a weight ratio of 3:7, followed by corona poling to align the chromophores. The optimal poling parameters for maximizing electro–optic activity were as follows: a poling temperature of 80°C, a poling voltage of 1.8 kV, and a poling duration of 30 min (Figure 5B). The resulting guest–host nonlinear polymer CLD‐1/PMMA with a thickness of 1 µm exhibited a high electro–optic coefficient of approximately 78.9 pm/V.

On the basis of electro–optic modulation theory, a three‐dimensional electro–optic coupling model was developed in COMSOL to design the waveguide structure and electrode parameters for the photonic MZM ECG sensor. The travelling‐wave electrode has a coplanar waveguide structure made of gold (conductivity: 4.26 × 10^7^ S/m), and the dielectric constant of the PDMS substrate is 2.75. To achieve a characteristic impedance of approximately 50 Ω, the central electrode width was set to 35 µm, the electrode spacing was set to 2.3 µm, and the electrode thickness was set to 200 nm. The bent waveguide structure was optimized, resulting in the following final parameters: a curvature radius of 1750 µm, a lateral deflection angle of 0.02 rad, and a modulation arm length of 8000 µm (Figure 5C). The results demonstrated that the photonic MZM ECG sensor can achieve a π phase shift under a driving voltage of 1.5 V, thereby effectively reducing the driving voltage and increasing the modulation sensitivity to weak ECG signals.

The half‐wave voltage and extinction ratio are two key performance parameters of the photonic MZM ECG sensor. A modulated electrical signal was generated by a function signal generator and applied to the electrodes of the MZM via a microwave probe, with a DC bias voltage ranging from 0 to 4 V in 0.1‐V increments. The change in the output light intensity was recorded using an optical power meter to determine the MZM modulation response. The measured half‐wave voltage was approximately 1.6 V, and the extinction ratio was 12.06 dB (Figure 5D). SEM images and sample chip images of the photonic MZM ECG sensor are shown in Figure 5E.

### 2.6 Photonic MZI Sweat Glucose Sensor Design and Characterization

Unlike the previously mentioned MZM employed in ECG‐sensing photonic sensors, the photonic MZI sweat glucose sensor features a similar waveguide structure, but it omits the modulation electrodes and does not rely on an external electrical signal for phase modulation. Instead, the sensor provides refractive index sensing through a sensitive layer applied to the sensing arm.

**FIGURE 6:**
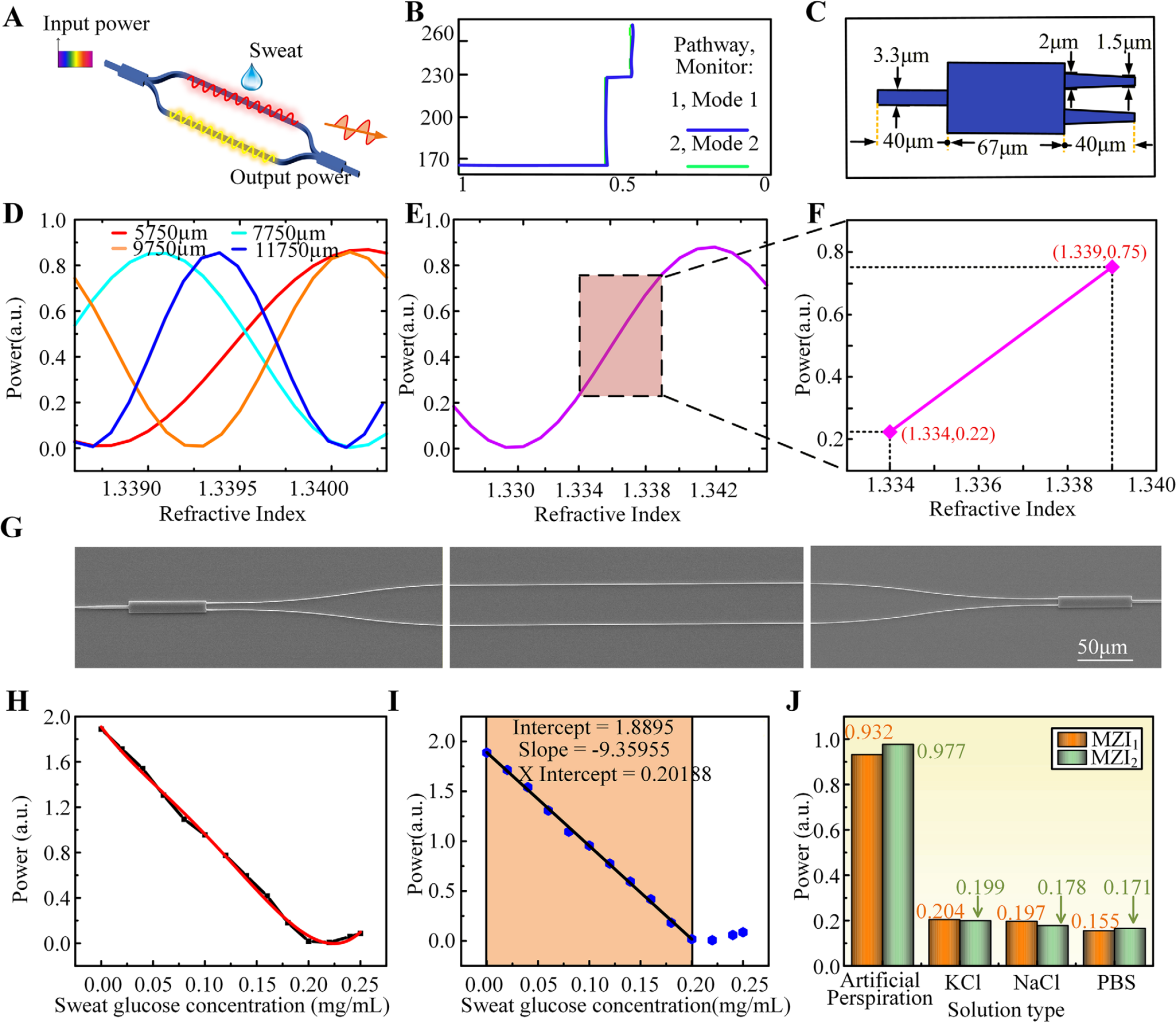
Simulation and test results for the photonic MZI sweat glucose sensor. (A) Schematic illustration of the photonic MZI sweat glucose sensor. (B) Energy distribution at the two output ports of the MMI coupler. (C) Structural parameters of the MMI coupler. (D) Relationship between arm length and the refractive index. (E) Relationship between the optical output power and refractive index at a sensing arm length of 7310 µm. (F) Simulation curve of the sensor in the linear region. (G) SEM image of the photonic MZI sweat glucose sensor. (H) Relationship between the sweat glucose concentration and optical output power. (I) Test curve of the sensor in the linear region. (J) Specificity test of the photonic MZI sweat glucose sensor.

While sweat offers a promising noninvasive alternative for glucose monitoring, its application remains limited because of the moderate correlation between sweat and blood glucose concentrations [40, 41, 42]. However, the glucose level in sweat is much lower than that in blood. To accurately detect such a low concentration of glucose in sweat, the MZI‐based sweat glucose sensor was designed to be highly sensitive. For refractive index sensing applications, a window was opened on the sensing arm and covered with an immobilized GOD‐sensitive layer, allowing for the evanescent field of the guided mode to directly interact with glucose in sweat. When glucose in sweat experiences a specific catalytic reaction with the enzyme, the local refractive index changes, thereby altering the effective refractive index of the guided mode in the sensing arm. This, in turn, causes a change in the difference in optical path between the two arms (Figure 6A). As a result, the output interference light power increases monotonically with increasing glucose concentration, facilitating quantitative measurement. To optimize the sensor performance, we determined the waveguide geometric parameters, and the MMI coupler achieved a 1:1 optical power split (Figure 6B); the specific structural parameters are shown in Figure 6C. When the refractive index changed from 1.334 to 1.339, the output optical power increased from 0.22 to 0.75 mW. The fitting curve for sweat glucose was *y =* 106.27041x − 141.54587, with a sensitivity of 106.270 pm/RIU and *R^2^ =* 1 (Figure 6D–F), indicating that the photonic MZI sweat glucose sensor is highly sensitive for measuring low concentrations of glucose in sweat. SEM images of the photonic MZI sweat glucose sensor are shown in Figure 6G.

In the experiments, GOD was firmly immobilized on the surface of the MZI sensing arm via covalent coupling using a silane coupling agent. Through the use of a 10‐mW input light source, the relationship between the output optical power of the photonic MZI sweat glucose sensor and the glucose concentration was determined. A linear response was observed in the range of 0–0.2 mg/mL, with a sensitivity of 9.3595 mW/(mg/mL) in the linear region (Figure 6H,I). To prevent residual interference from solutions of different concentrations, the sensing arm was rinsed with deionized water before each test. The light intensity responses of the photonic MZI sweat glucose sensor to three distinct solutions (PBS, NaCl, and glucose) were subsequently compared. As shown in Figure 6J, the sensor exhibited the greatest output power change in the glucose solution, reaching 0.932 mW, whereas the changes under KCl, PBS, and NaCl conditions reached only 0.204, 0.155, and 0.197 mW, respectively. These results indicate that the GOD immobilized on the surface of the MZI sensing arm can specifically recognize glucose.

### 2.7 AWG Design and Characterization

**FIGURE 7:**
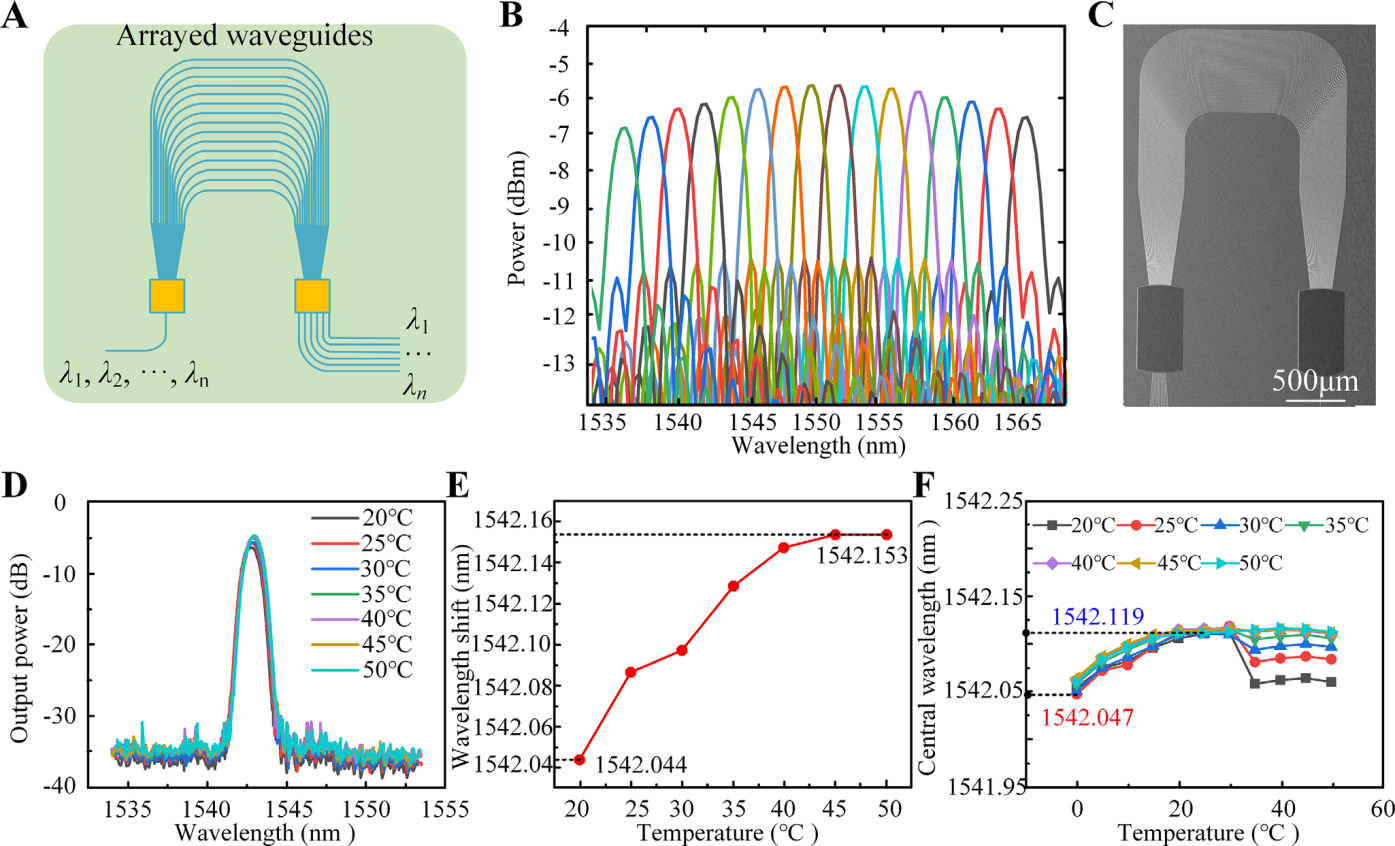
Schematic illustration and SEM images of the AWG. (A) Schematic structure of the 1×16 AWG. (B) Transmission spectrum of the AWG. (C) SEM image of the AWG. (D) Variation in the AWG central wavelength with temperature (20°C to 50°C). (E) Central wavelength shift of the AWG. (F) Temporal evolution of the AWG central wavelength under different constant temperatures (recorded every 5 min for a duration of 30 min).

An arrayed waveguide grating can separate or combine signals with different wavelengths. An AWG unit was employed to separate and demodulate the reflection spectra of all the photonic WBG sensors. The structure of the AWG is shown in Figure 7A. The unit images the field in an input waveguide onto an array of output waveguides in such a way that the signals of different wavelengths present in the input waveguide are imaged onto different output waveguides. In this study, WDM‐Phasar software was adopted for modelling and simulation; on the basis of the center wavelengths of the photonic WBG sensors and their expected spectral shifts, the final 1 × 16 AWG design has a waveguide width of 2 µm and incorporates 64 arrayed arms. The initial arm length is 1500 µm, with a path‐length increment of 32.289 µm and an angular spread of 30.202°. As shown in Figure 7B, the center wavelengths of the sixteen output channels are 1536.2, 1538.2, 1540.2, 1542.1, 1544.1, 1546.0, 1548.0, 1549.8, 1551.6, 1553.6, 1555.4, 1557.4, 1559.3, 1561.3, 1563.2, and 1565.2 nm. SEM images of the AWG are shown in Figure 7C.

To quantitatively evaluate the temperature stability of the AWG, a broadband light source was used to inject an optical signal with a wavelength of 1550 nm and an optical power of 10 mW into the packaged AWG. A high–low temperature chamber was employed to precisely control the ambient temperature, and an optical spectrum analyzer (OSA) was used to record the spectral shift of the output light (Figure 7D). As presented in Figure 7E, within the temperature range of 20°C to 50°C, the central wavelength of the AWG channel varied from 1542.04 to 1542.15 nm, with an overall wavelength fluctuation of only 0.11 nm. Such a small wavelength drift is negligible and has little influence on the experimental results. In addition, the temporal stability of the AWG under constant temperature conditions was further investigated. The ambient temperature was set to 20°C, 25°C, 30°C, 35°C, 40°C, 45°C, and 50°C. Each temperature was maintained steadily for 50 min, and the central wavelength of the output spectrum was recorded every 5 min, as illustrated in Figure 7F. The results indicate that the maximum central wavelength fluctuation of the AWG was only 0.072 nm across the tested temperature range, demonstrating long‐term time‐domain stability.

## 3 Experimental Results and Analysis

**FIGURE 8:**
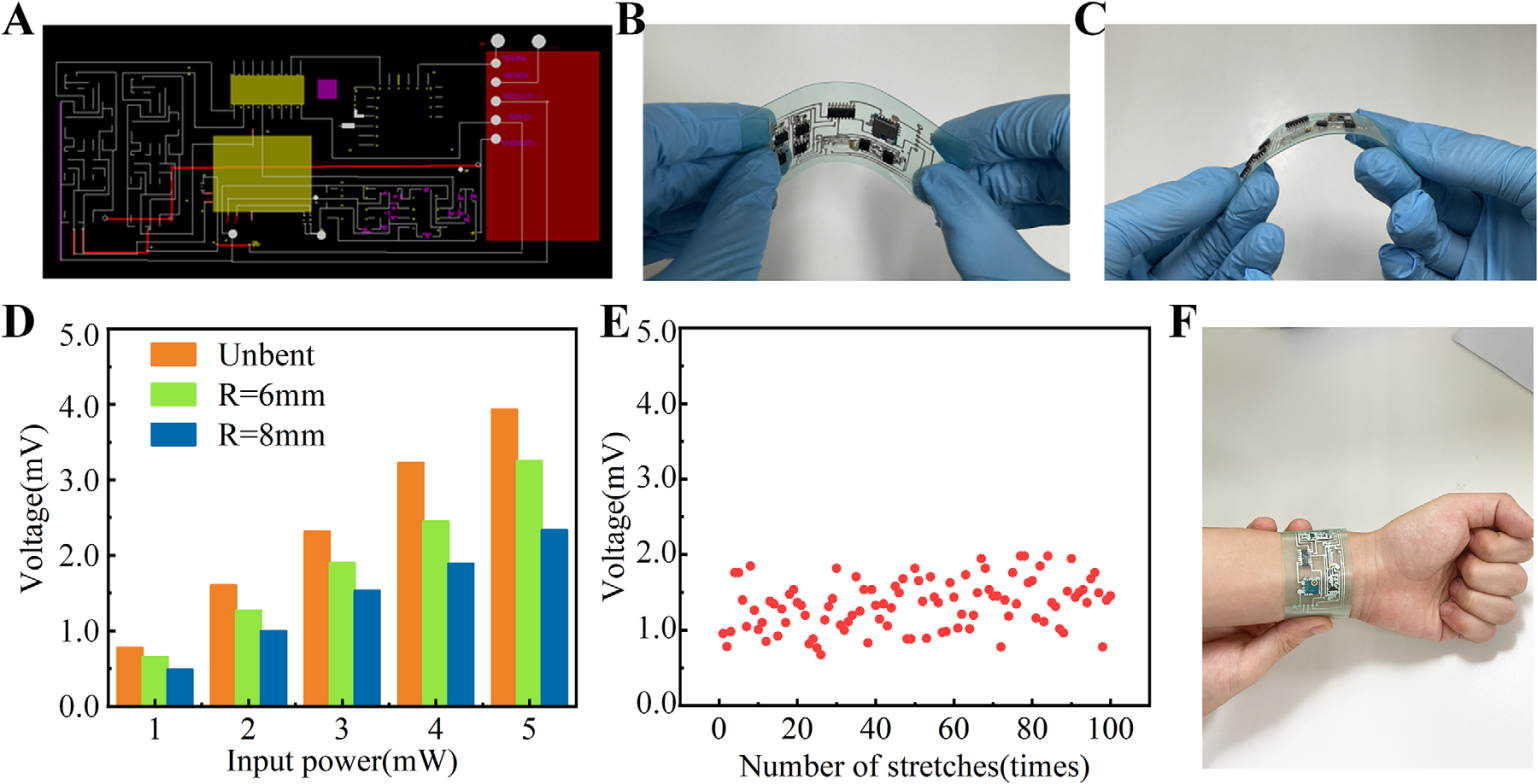
Mechanical flexibility and deformation performance evaluation of the flexible photonic chip. (A) Schematic illustration of the designed flexible PCB (approximately 6 cm × 2.5 cm). (B,C) Photographs of the as‐prepared flexible PCB under bending, twisting, and stretching deformations. (D) Comparison of the voltage signal amplification under different bending conditions: flat state, bending radius of 6 mm, and bending radius of 8 mm. (E) Experimental results of 100 stretching cycles of the flexible PCB. (F) The flexible PCB wristband supports pulse wave monitoring.

The flexible photonic chip developed in this study is approximately 1 cm × 0.5 cm in size. In practical wearable tests, the chip is attached to gently curved parts of the human body, such as the forehead, chest, and wrist. Both the polydimethylsiloxane (PDMS) and polymethyl methacrylate (PMMA) employed in this study are medical‐grade polymer materials with excellent biocompatibility and have been widely demonstrated to be suitable for wearable and skin‐contact biomedical applications [43, 44, 45, 46]. The deformation caused by daily physical activity is extremely limited and negligible and has little influence on the detection signals and experimental results. To systematically evaluate the mechanical flexibility and surface adaptation capability of the chip, a DB100 microelectronic printer was adopted for the fabrication and integration of a flexible printed circuit board (PCB). The designed circuit pattern is illustrated in Figure 8A. With the PDMS film serving as the flexible substrate and the SP1168 conductive silver paste as the printing material, the major fabrication procedures include pattern design, printing parameter optimization, PDMS surface modification, precision circuit printing, component mounting, and curing and baking. The as‐prepared flexible integrated circuit can stably withstand multiple mechanical deformations, including bending, twisting, and stretching, demonstrating excellent deformation adaptability (Figure 8B,C).

To further investigate the effect of bending deformation on the optoelectronic performance of the chip, comparative tests were conducted under three bending conditions: flat state and bending radii of 6–8 mm. As presented in Figure 8D, the output signal exhibits only a slight attenuation with increasing bending radius, while all the signal values remain within the normal working range. These results verify that the chip can maintain a stable and continuous signal output under the typical curvature of human skin attachment. In addition, 100 cyclic stretching tests were carried out to evaluate the long‐term operational reliability. After repeated mechanical loading, the flexible PCB still operates normally without obvious performance degradation or structural damage, which confirms its excellent mechanical flexibility and fatigue resistance (Figure 8E). Owing to its outstanding flexibility, the flexible PCB can closely fit the wrist skin and effectively collect pulse signals (Figure 8F).

In the experiments, the reflected signals from the photonic WBG blood glucose sensors, photonic WBG pressure sensor array, and photonic WBG temperature sensors were all wavelength‐demodulated by the AWG, and the output light intensity at each channel port was measured. To demodulate the WBG reflection spectrum, the relative intensity demodulation method was employed. In this approach, two adjacent output channels of the AWG are used to demodulate the wavelength of an individual WBG. Let the central wavelength of the WBG be denoted as *λ_WBG_* and the central wavelengths of the *m_th_* and *(m+1)_th_* channels of the AWG be denoted as *λ_m_* and *λ_m+1_*, respectively, with their corresponding output powers denoted as *P_m_* and *P_m+1_*, respectively. When a shift in the central wavelength of the WBG reflection, i.e., *λ_WBG_*, occurs because of external factors such as glucose concentration or pressure, the output powers *P_m_* and *P_m+1_* of the adjacent channels change accordingly. The logarithm of the ratio of these two powers is defined as the wavelength demodulation function, *ρ(λ)*, which is ln*(P_m+1_/P_m_)*.

**FIGURE 9:**
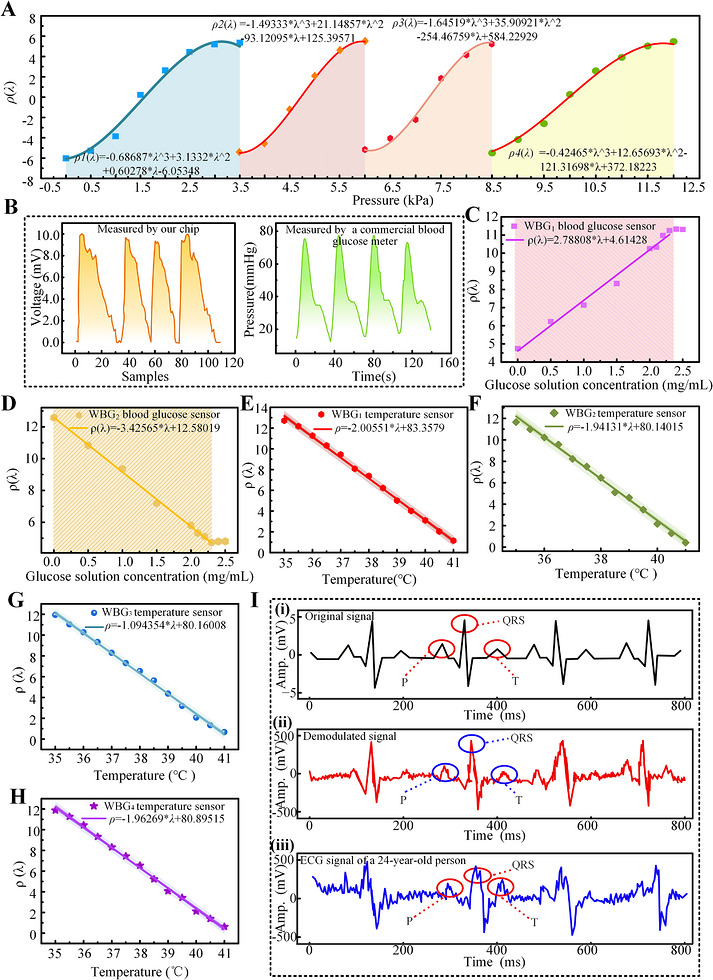
Demodulation experiments of the integrated photonic chip. (A) Demodulation curve of the photonic WBG pressure sensor under pressures ranging from 0–12 kPa. (B) Waveform of the pulse after filtering. (C) Demodulation curve of the photonic WBG_1_ blood glucose sensor. (D) Demodulation curve of the photonic WBG_2_ blood glucose sensor. (E) Demodulation curve of the photonic WBG_1_ temperature sensor at 35–41°C. (F) Demodulation curve of the photonic WBG_2_ temperature sensor at 35–41°C. (G) Demodulation curve of the photonic WBG_3_ temperature sensor at 35–41°C. (H) Demodulation curve of the photonic WBG_4_ temperature sensor at 35–41°C. (I) Experimental results of ECG measurements. (i) Standard ECG signal; (ii) signal recorded by the MZM device; (iii) ECG waveform collected from a volunteer.

In the pressure measurement experiment, pressure was gradually applied to the sensor array, with the value increasing from 0 to 12 kPa, and changes in the AWG output optical power were recorded. The output channel intensities were demodulated by channels 8 and 9 of the AWG in the range of 0–3.5 kPa, by channels 9 and 10 for 3.5–6 kPa, by channels 10 and 11 for 6–8.5 kPa, and by channels 11 and 12 for 8.5–12 kPa. The results indicated that with increasing external pressure, the output power curve exhibited segmented sensitivity characteristics. Demodulation curves were obtained for pressure ranges of 0–3.5, 3.5–6, 6–8.5, and 8.5–12 kPa (Figure 9A). To demonstrate the feasibility of pulse monitoring, the proposed pressure sensor array was attached to the radial artery at the wrist, and the time‐domain pulse waveform was recorded, as shown in Figure 9B. The system continuously acquired voltage signals at a sampling interval of 10 ms. After data processing, optical signal waveforms that clearly reflected the physiological characteristics of the human pulses were finally obtained. To further evaluate the detection performance of the developed device, synchronous comparative tests were conducted using a commercial pulse oximeter (CMS50E, Contec). Comparative analysis demonstrates that the pulse waveforms measured by the proposed integrated photonic chip are consistent with those collected by the commercial device. These results fully confirm the high detection accuracy and operational stability of the integrated photonic chip for real‐time human pulse monitoring, which provides strong support for its practical application.

In the blood glucose measurement experiment, glucose solutions with concentrations ranging from 0 to 2.0 mg/mL were prepared at intervals of 0.5 mg/mL. Within the range of 2.0–2.5 mg/mL, a concentration interval of 0.1 mg/mL was adopted. Glucose solutions of varying concentrations were sequentially applied to the blood glucose WBG sensing area. The linear device of the blood glucose WBG sensor demonstrates a good linear response within the concentration range of 0–2.3 mg/mL (Figure 9C,D). In the temperature measurement experiment, the reflection wavelengths from the four WBGs were output through the sixth, fifth, second, and third channels of the AWG. In the experiment, the sensors were placed on a heating stage, and the temperature was increased from 35°C to 41°C in 0.5°C increments. The experimental results are shown in Figure 9E–H. The demodulation curves of all four WBG sensors exhibited excellent linearity within the target measurement range. To verify the performance of the ECG measurements, we first performed a comparative test using a standard signal (amplitude of 5.5 mV) output from an ECG simulator, as shown in Figure 9I‐i,ii. The demodulated results reveal clear and distinct QRS complex features. In vivo measurements were subsequently carried out on a healthy volunteer, and the results are presented in Figure 9I‐iii. The characteristics of normal human ECG monitoring waveforms, such as QRS, P‐ and T‐waves, are clear and significant.

## 4 Discussion

PDMS/PMMA is inherently sensitive to environmental variations, as the relatively high thermo–optic coefficient and thermal expansion coefficient of these materials may induce additional Bragg wavelength drift under ambient temperature fluctuations and localized body temperature changes. Such effects could influence the accuracy and long‐term calibration stability of photonic WBG sensors for glucose, pressure, and temperature monitoring. In addition, long‐term operation may lead to polymer ageing, molecular relaxation, and moisture absorption, thus gradually altering the effective refractive index of the waveguide. Moreover, restricted by the technical conditions of the ICP etching process, the actual cross‐sectional morphology of the WBG grating deviates from the ideally designed rectangular ridge structure, presenting a nonrectangular profile in the SEM images. Such fabrication deviation may also affect the optical consistency of the device. Therefore, future work should focus on developing polymer materials with low thermal response and low hygroscopicity, optimizing micro/nanofabrication processes to improve pattern fidelity, and adopting nonthermal structural designs such as dual material compensation or stress compensation cladding layers, to increase environmental robustness and operational stability.

Furthermore, the stability and repeatability of the glucose oxidase immobilization layer remain critical factors influencing biosensing reliability in both the photonic WBG glucose sensor and the photonic MZI sweat glucose sensor. Enzyme activity degradation during prolonged use or under elevated temperatures may reduce sensitivity and linearity. Improved immobilization strategies, nanostructure‐enhanced interfaces, or enzyme‐free sensing mechanisms could improve the long‐term performance. At the system level, existing validations were based mainly on in vitro and short‐duration experiments. Comprehensive in vivo studies under dynamic physiological conditions, including motion, sweating, and skin deformation, are necessary to evaluate system robustness and promote the translation of the integrated photonic chip towards practical wearable health monitoring applications.

## 5 Conclusions

In this study, we successfully developed a flexible photonic chip based on a polymer material (PDMS/PMMA) with a size of only 1cm × 0.5 cm. The experimental results demonstrated that the photonic WBG temperature sensor enables accurate temperature monitoring within the range of 35–41°C. The photonic WBG blood glucose sensor achieved stable measurements within the concentration range of 0–2.3 mg/mL. The photonic MZI sweat glucose sensor provided a detection range of 0–0.2 mg/mL and a sensitivity of approximately 9.3595 mW/(mg/mL), indicating favorable linearity. The photonic MZM ECG sensor could effectively demodulate weak ECG signals with a half‐wave voltage of 1.6 V. Based on on‐chip multiwavelength channel management and optical path collaborative optimization, the proposed integrated photonic chip offers a compact, integrated, and highly sensitive platform for real‐time detection of multiple vital signs and metabolic signals, which could revolutionize personalized health monitoring.

## 6 Methods

### 6.1 Device Fabrication and Key Process Parameters

The device is fabricated monolithically on an ITO‐coated glass substrate based on a PDMS/PMMA hybrid polymer system. The overall process consists of eight key steps: spin‐coating the PDMS bottom cladding → spin‐coating the CLD‐1/PMMA electro‐optic layer → photolithography and ICP etching of the waveguide core layer → spin‐coating the PDMS top cladding → magnetron sputtering of metal electrodes → electrode patterning via a lift‐off process → ICP etching of the top cladding in the grating region → final release of the chip.

The detailed fabrication process is as follows. First, the PDMS precursor and curing agent are mixed at a weight ratio of 10:1, spin‐coated in two steps at 500 rpm for 10 s and 3500 rpm for 30 s, and cured at 150°C for 15–20 min to form bottom cladding with a thickness of approximately 10 µm. A 30 wt.% CLD‐1/PMMA electro‐optic core layer is subsequently spin‐coated under two‐step conditions of 500 rpm for 10 s and 1000 rpm for 30 s, yielding an ∼1 µm‐thick film after curing. The waveguide structures are patterned via ultraviolet photolithography, metal masking, and ICP etching, followed by removal of the metal mask using acetone.

The PDMS top cladding is then spin‐coated under the same parameters to a thickness of ∼10 µm. Metal electrodes are deposited by magnetron sputtering combined with the lift‐off technique: a 20 nm Cr adhesion layer and a 200 nm Al electrode layer are sequentially sputtered, with AZ‐5214 photoresist serving as the patterning mask. In the sensing regions, the upper PDMS cladding is selectively etched via ICP using an SF_6_:O_2_ gas mixture of 15:3, a chamber pressure of 0.5 Pa, and an RF power of 250 W to ensure smooth sidewalls and high etching precision.

## Author Contributions

**X. L**., **H. L**., and **M. H**. designed the outline of this manuscript. **H. L**., **X. L.,** and **M. D**. were responsible for writing the initial manuscript and depicting all the figures and tables. **X. L**., **Z. N**., **D. L**., **L. Y**., **L. Z**., and **L. C**. provided technical assistance, helped to collect clinical samples, reviewed the manuscript, and amended the reference. **F. H.‐R**. and **E. L**. revised the manuscript. All authors have read and approved the article.

## Ethics Statement

We hereby confirm that this study involving human participants was conducted in accordance with the guidelines of the Ethics Committee of Tianjin Chest Hospital, with formal approval granted (Approval No. 2025YS‐074‐01). Informed consent was obtained from all participants prior to the study.

## Conflicts of Interest

The authors declare no conflicts of interest.

## Data Availability

The data that support the findings of this study are available on request from the corresponding author. The data are not publicly available due to privacy or ethical restrictions.
